# Land Management and Microbial Seed Load Effect on Rhizosphere and Endosphere Bacterial Community Assembly in Wheat

**DOI:** 10.3389/fmicb.2019.02625

**Published:** 2019-11-15

**Authors:** Vanessa Nessner Kavamura, Rebekah J. Robinson, Rifat Hayat, Ian M. Clark, David Hughes, Maike Rossmann, Penny R. Hirsch, Rodrigo Mendes, Tim H. Mauchline

**Affiliations:** ^1^Sustainable Agriculture Sciences, Rothamsted Research, Harpenden, United Kingdom; ^2^Plant Pathology Laboratory, RHS Garden Wisley, Woking, United Kingdom; ^3^Department of Soil Science and Soil and Water Conservation, Pir Mehr Ali Shah Arid Agriculture University, Rawalpindi, Pakistan; ^4^Computational and Analytical Sciences, Rothamsted Research, Harpenden, United Kingdom; ^5^Laboratory of Environmental Microbiology, Embrapa Meio Ambiente, Jaguariúna, Brazil

**Keywords:** wheat, microbiome, rhizosphere, endosphere, seed, embryo

## Abstract

Microbial community ecology studies have traditionally utilized culture-based methodologies, though the advent of next-generation amplicon sequencing has facilitated superior resolution analyses of complex microbial communities. Here, we used culture-dependent and -independent approaches to explore the influence of land use as well as microbial seed load on bacterial community structure of the wheat rhizosphere and root endosphere. It was found that niche was an important factor in shaping the microbiome when using both methodological approaches, and that land use was also a discriminatory factor for the culture-independent-based method. Although culture-independent methods provide a higher resolution of analysis, it was found that in the rhizosphere, particular operational taxonomic units (OTUs) in the culture-dependent fraction were absent from the culture-independent fraction, indicating that deeper sequence analysis is required for this approach to be exhaustive. We also found that the microbial seed load defined the endosphere, but not rhizosphere, community structure for plants grown in soil which was not wheat adapted. Together, these findings increase our understanding of the importance of land management and microbial seed load in shaping the root microbiome of wheat and this knowledge will facilitate the exploitation of plant–microbe interactions for the development of novel microbial inoculants.

## Introduction

Microbes are fundamental for maintenance of life on Earth and it is well known that microbial communities in soil influence plant health, growth, and resource use efficiency, especially the subset that is recruited by plants to form the root microbiome (Berendsen et al., 2012; Mendes et al., 2013). Beneficial microbes have been isolated from crop plants for many years, though limitations in the ability to readily culture the majority of members of the plant microbiome has hampered our understanding of their community dynamics. It is clear that microbes have a potential role to play in the sustainable intensification of agriculture, though the tractability of their isolation and use has not yet been optimized.

Recent advances in next-generation sequencing has allowed unprecedented studies in soil microbial communities. These studies have revealed that pH is a primary driver of bulk soil community structure (Fierer and Jackson, 2006). Additonally, it has been shown that the rhizosphere is the most complex root associated community, followed by the rhizoplane and root endosphere the simplest (Bulgarelli et al., 2012; Lundberg et al., 2012). Other studies have investigated the importance of plant genotype on community selection, and it has been shown that there are a number of changes in bacterial taxa abundance driven by plant species (Bulgarelli et al., 2013), and to a lesser extent, cultivar (İnceoğlu et al., 2012; Winston et al., 2014; Mauchline et al., 2015). Other work has investigated the role of land management in agricultural systems on the soil microbiome. It has been found that application of agrochemicals such as nitrogen fertilizers influence both the bulk soil and rhizosphere microbiome (Kavamura et al., 2018), and other studies have examined the role of physical land management of bulk soil (Lumini et al., 2011; Sengupta and Dick, 2015), although relatively little work has examined how these processes influence the plant root microbiome. Transmission of microbes via seeds is also a relevant factor to be considered because it can impact the composition of the plant microbiome, with consequences for plant productivity (Shade et al., 2017). However, links between seed and soil microbiomes are not yet fully understood (Nelson et al., 2018).

Here, we examine the wheat plots at the Rothamsted Highfield experiment and investigate the relative importance of land use (continuous wheat compared to grassland to wheat and bare fallow to wheat conversions) on the bulk soil, rhizosphere, and root endosphere community selection. Unlike most studies which mainly use culture-independent methods to investigate the roles of certain variables on microbial communities, we compared two amplicon sequencing approaches: “total community” with a novel plate culture wash extraction for soil and agriculture systems, with the aim of establishing the level of discrimination that each method allows. In addition, we assessed the impact of microbial seed load on culturable bacterial communities from excised embryos and complete wheat seeds for the recruitment of rhizosphere and endosphere communities, hypothesizing that seed load is important for the assembly of the rhizosphere and endosphere wheat microbiome.

## Materials and Methods

### Plant and Soil Sampling

The Highfield experiment is located at the Rothamsted Research farm in Harpenden, Hertfordshire, United Kingdom. The site had been under pasture for centuries when, in 1949, sections were switched to continuous arable (wheat) cultivation. In 1959, further sections of grassland were converted to a bare fallow treatment in which weeds are regularly removed. In October 2008, 10 × 6 m areas within the existing bare fallow, arable, and grassland sections were converted to one of the alternative treatments in a randomized block design to provide three plots for each permanent treatment (i.e., grassland, arable, or bare fallow) and three plots for each conversion treatment (i.e., grassland to bare fallow, grassland to arable, arable to grassland, arable to bare fallow, barefallow to arable, and bare fallow to grassland) resulting in a total of 9 treatments and 27 plots (Hirsch et al., 2017). Wheat plants, cultivar Hereward, were sampled from the nine plots under arable cultivation in July 2012 at growth stage 69 (late flowering). From each plot, five plants were sampled in a “W” formation across the plot using a hand trowel, with the crown roots and a proportion of the primary root, seminal, and lateral roots attached. Plants were placed in plastic bags and transported to the laboratory for processing. Bulk soil was sampled in October 2011 (pre-season) and prior to the following crop cycle, in February 2013 from these nine arable plots in a “W” formation across the plot to a depth of 25 cm using a 3 cm diameter corer. Five cores per plot were pooled and mixed prior to sieving through a 2 mm mesh. Each plot sample consisted of five plants or soil cores which were pooled together and considered as one replicate, with a total of three replicates (plots) per treatment. A portion of the total bulk soil sample (20 g) was then frozen at 80°C prior to DNA extraction and the remainder kept at 4°C prior to microbial culture. The experimental design consisted of three types of soil management [continuous arable (AA), bare fallow to arable (BA) or grassland to arable (GA)] × 1 niche (rhizosphere) × 3 replicates (plots) for each management system, collected once in 2012, with a total of nine samples and the same three land management system × 3 replicates × 2 bulk soil sampling times (2011 and 2013), a total of 18 samples (Supplementary Table S1).

### “Seed–Embryo” Experiment

**FIGURE 1:**
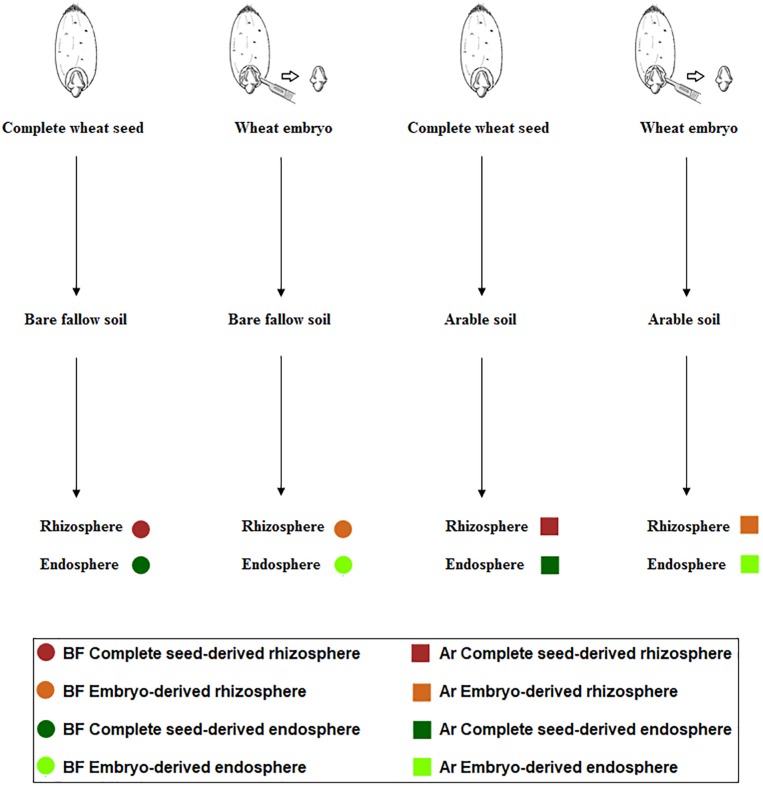
Scheme representing the setup of the experiment to recover culturable rhizosphere (brown) and endosphere (green) bacteria from complete wheat seeds (dark color) and embryos (light color) in arable soil (square) and bare fallow soil (circle).

The experimental design is summarized in Figure 1. The aim of this experiment was to ascertain the influence of soil management history and microbial seed load in shaping the root microbiome. We chose bare fallow soil and continuous arable soil as contrasting soil management types and cultured wheat plants derived from two seed types: complete seeds or microbiome-free embryos (Robinson et al., 2016b).

In June 2013, a further sampling of bulk soil from the bare fallow and continuous arable plots was made using a small hand trowel in a W formation across each plot. Soil for each treatment from all three plots was pooled and thoroughly mixed and subsequently sieved as described above. This resulted in pooled bulk soil and pooled arable soil samples. Prior to sowing, wheat seeds (cultivar Cadenza) were surface sterilized following the protocol of Robinson et al. (2016a) and left for overnight imbibition in sterile water at 4°C. Next, a proportion of the wheat embryos were carefully and aseptically excised, as described by Robinson et al. (2016b). Fifteen pots (13 cm) were filled with arable soil and a further 15 with bare fallow soil which were allowed to equilibrate in the glass house for 1 week. For each soil management type, six pots were planted with single seeds, six planted with single excised embryos, and three bulk soil pots remained unplanted. Pots were incubated in the glasshouse at 20°C with a 16-h per day light regime, and were watered daily with tap water. Any weeds germinating in the pots were removed by hand. Plants were harvested at the start of flowering stage (Zadoks growth stage 61), at approximately 10 weeks after sowing, and rhizosphere and endosphere processing performed. Bulk soil samples were taken after 10 weeks, at the same time as rhizosphere sampling was performed. Experimental design consisted of two types of original seed (either complete seed or embryo) × 2 soil managements (arable or bare fallow) × 2 niche (endosphere or rhizosphere) × 6 plants (replicates) = 48 samples, plus three additional bulk soil replicates from each soil management, a total of 54 samples (Supplementary Table S1).

### Rhizosphere Processing

Loose soil was shaken from each plant and discarded before cutting the root systems into 2–3 cm sections and mixed by shaking in a bag. A 10 g sub-sample was transferred to a 50 ml Falcon tube and 30 ml sterile water added. The roots were vortexed at high speed for 90 s to release the rhizosphere soil from the root system. The roots were placed in a separate tube for endophyte work. The remaining rhizosphere soil suspensions were centrifuged at 4,000 rpm for 10 min at 4°C. After this time the supernatant was discarded and the soil frozen at −80°C prior to DNA extraction. Prior to freezing, 1 g rhizosphere soil was used to prepare a serial dilution series, of which 100 μl of the 10^–4^ dilution was plated onto 1/10th TSA agar Petri plates (Oxoid) and incubated at 27°C for 7 days. After this time agar plates were flooded with 3 ml of sterile water, and a sterile glass spreader was used to resuspend all colonies on a given plate. 1.5 ml of resuspended culture from each plate was then transferred to a sterile 1.5 ml microfuge tube and spun at 16,000 rpm for 5 min. After this time the supernatant was removed and the remaining culture subjected to DNA extraction. For isolation of rhizosphere bacteria, experimental design consisted of three soil management types [continuous arable (AA), bare fallow to arable (BA), or grassland to arable (GA) × 1 niche (rhizosphere) × 3 replicates (plots) for each management system, a total of nine samples (Supplementary Table S1).

### Isolation of Wheat Endophytes

Endosphere isolates were recovered according to the method described by Robinson et al. (2016a). Briefly, roots were twice vortexed in sterile distilled water (SDW) before sterilization using an optimized 16-min surface sterilization procedure with agitation in sodium hypochlorite solution (1.6% active chlorine), a rinse in SDW, a 1-min wash in 95% ethanol, followed by three rinses in SDW with agitation. For plants harvested in the “seed–embryo excision” experiment a shorter sodium hypochlorite sterilization period of 10 min was adopted as the 16 min period optimized for field grown plants was found to be too harsh, and killed the entire root microbiome of pot grown plants. Following sterilization, 1 cm was discarded from the ends of each sample to remove tissue which may have been affected through bleach penetration by capillary action. Fresh tissue samples were weighed and 1 ml SDW was added for every 0.1 g tissue. Samples were completely macerated in SDW using a sterile pestle and mortar, diluted a further 100fold, and 100 μl plated onto a 1/10th TSA Petri plate and incubated at 27°C for 7 days. For isolation of endophytic bacteria, experimental design consisted of three soil management types [continuous arable (AA), bare fallow to arable (BA), or grassland to arable (GA)] × 1 niche (endosphere) × 3 replicates (plots) for each management system. One outlier was removed from the analysis, in a total of eight samples (Supplementary Table S1).

### Soil DNA Extraction and Quantitation

For each sample, DNA was isolated from 0.25 g of soil using the MoBio PowerSoil^TM^ DNA Isolation Kit (Carlsbad, CA, United States). Extractions were performed according to the manufacturer’s instructions but with the use of the MP Biomedicals FastPrep-24 machine for 30 s at 5.5 m/s and the resuspension of DNA in 100 μl sterile DNA-free PCR grade water. Genomic DNA concentration and purity was determined by NanoDrop spectrophotometry (Thermo Scientific, Wilmington, DE, United States) as well as with a Qubit 2.0 Fluorimeter and dsDNA HS assay kit (Thermo Fisher).

### Mixed Culture DNA Extraction and Quantitation

Each sample was subjected to Sigma GenElute Bacterial Genomic DNA extraction kit using the lysozyme utilizing Gram-positive bacterial preparation method to ensure lysis of both Gram-positive and Gram-negative bacterial cells. The protocol was followed according to the manufacturer’s instructions and DNA was resuspended in 200 μl sterile DNA-free PCR grade water. Sample genomic DNA concentration and purity were determined as above.

### Illumina Bacterial 16S rRNA Gene Sequencing

The bacterial 16S rRNA gene was amplified from culture-dependent bulk soil, endosphere and rhizosphere DNA samples, as well as culture-independent bulk and rhizosphere soil DNA samples, using barcoded universal prokaryotic primers 515F (5′-GTGCCAGCMGCCGCGGTAA-3′) and 806R (5′-GGACTACHVGGGTWTCTAAT-3′) for paired-end microbial community analysis (Caporaso et al., 2011) targeting the V4 region and subjected to Illumina^®^ sequencing using the MiSeq platform to generate 2 × 150 bp paired-end reads at the high-throughput Genome Analysis Core (HGAC), Argonne National Laboratory (Illinois, United States).

### Sequence Analysis Pipeline

16S rRNA gene sequences were analyzed using the pipeline proposed by the Brazilian Microbiome Project (BMP) available at http://brmicrobiome.org (Pylro et al., 2014), with a few modifications. It uses Quantitative Insights Into Microbial Ecology (QIIME) (version 1.8.0) (Caporaso et al., 2010) and USEARCH 9.0^1^ (Edgar, 2010). Operational taxonomic units (OTUs) were defined to 97% sequence identity against SILVA 128 database (Quast et al., 2012). OTU data were transformed into relative proportions and significant differences in bacterial community structure were investigated by Permutational Analysis of Variance (PERMANOVA, Anderson, 2001) in Paleontological Statistics Software Package for Education and Data Analysis (PAST) (Hammer et al., 2001). PCoA plots and Analysis of Similarities (ANOSIM, Clarke, 1993) values were obtained using the same software. Bray–Curtis index was used for data obtained with culture-independent method whereas Jaccard index was used for data obtained with culture-dependent method. The online tool for comprehensive statistical, visual, and meta-analysis of microbiome data called MicrobiomeAnalyst (Dhariwal et al., 2017) was used for detecting OTUs which were differentially abundant among different treatments. The filtered OTUs were arranged in the required format and uploaded with the mapping and taxonomy files. Low abundance and low variance OTUs were removed using default values, where OTUs with less than two counts in <20% of the samples and 10% of the values below the determined inter-quantile range (IQR) were removed. The OTU table was normalized using the method of rarefying with replacement and relative log-expression (RLE) transformed, followed by DESeq2 tool which was used to evaluate differentially abundant taxa (expressed as log-transformed counts). Only OTUs assigned to Bacteria were used for Venn diagram construction using an online tool available at http://bioinformatics.psb.ugent.be/webtools/Venn/. For 16S rRNA gene amplicon analyses, each plot belonging to one land management system was considered as one replicate, with a total of three replicates per treatment.

## Results

### Culture-Independent Analysis: Land Management Shaping Bacterial Community Structure

**FIGURE 2:**
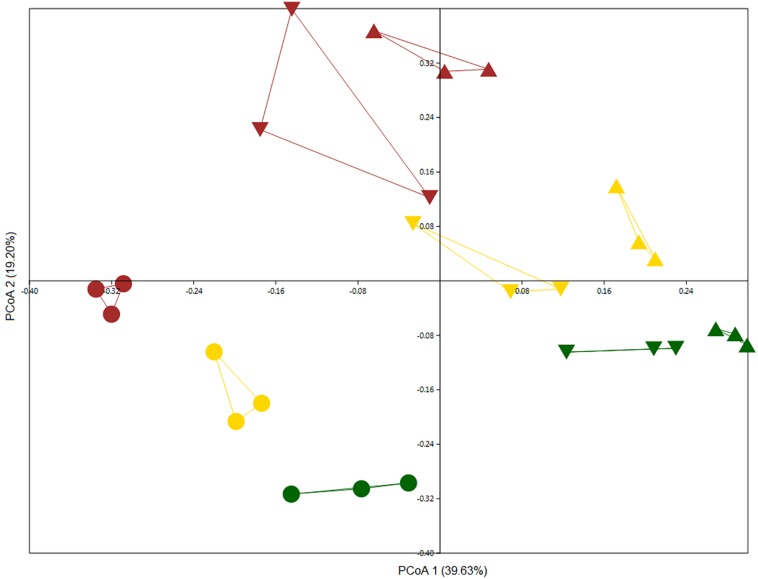
PCoA based on Bray–Curtis distance matrix was performed on culture-independent bulk soil samples collected in 2011 (triangle) and 2013 (inverted triangle) and rhizosphere samples (circle) collected in 2012 showing the structure of bacterial communities from the Highfield experiment under three types of land management: continuous arable (yellow), conversion of bare fallow to arable (brown), and conversion of grassland to arable (green).

We examined the microbiomes of bulk and rhizosphere soil samples for all wheat plots. We compared total community bulk soil samples from 2011 and 2013 with each other along with rhizosphere samples from 2012. Although bulk soil samples were more similar to one another than rhizosphere samples, they could be differentiated, indicating a possible temporal drift in bulk soil community structure (Figure 2) (two-way PERMANOVA, year: *F* = 7.615, *p* = 0.0001; land management: *F* = 7.011, *p* = 0.0001). The rhizosphere effect was the main discriminatory factor in shaping bacterial community, as rhizosphere samples clearly separated from bulk soil samples (Figure 2). In addition, land management also significantly influenced community structure (two-way PERMANOVA, niche: *F* = 10.305, *p* = 0.0001; land management: *F* = 5.0082, *p* = 0.0001). Regardless of land management, members of the phyla Acidobacteria, Actinobacteria, BRC1, Chloroflexi, FCPU426, Firmicutes, Latescibacteria, Nitrospirae, Omnitrophica, Planctomycetes, and Verrucomicrobia were significantly more abundant in bulk soil samples, whereas Bacteroidetes, Cyanobacteria, Deinococcus_Thermus, FBP, Fibrobacteres, and Proteobacteria were enriched in rhizosphere samples (Supplementary Figure S1).

The culture-independent analysis revealed that 60 OTUs were differentially abundant between land use treatments (Supplementary Figure S4). Forty-one OTUs were found to be significantly less abundant in samples from the conversion of grassland to arable and 19 were enriched for this treatment. Additionally, 21 OTUs were less abundant and 39 were enriched in the bare fallow to arable conversion. Finally, in the continuous arable treatment 5 OTUs were significantly less abundant and 55 OTUs were significantly enriched.

### Comparison of Culture-Independent and Culture-Dependent Methods in Assessing the Influence of Land Use in Wheat Rhizosphere Bacterial Community Structure

As expected, non-metric multidimensional scaling (NMDS) plots from culture-independent DNA samples could discriminate wheat communities according to previous land use (Figure 2) as confirmed by PERMANOVA analysis (*F* = 4.062, *p* = 0.0029). However, culture-dependent wheat rhizosphere bacterial communitities could not be discriminated based on land use (PERMANOVA, *F* = 0.944, *p* = 0.61).

**FIGURE 3:**
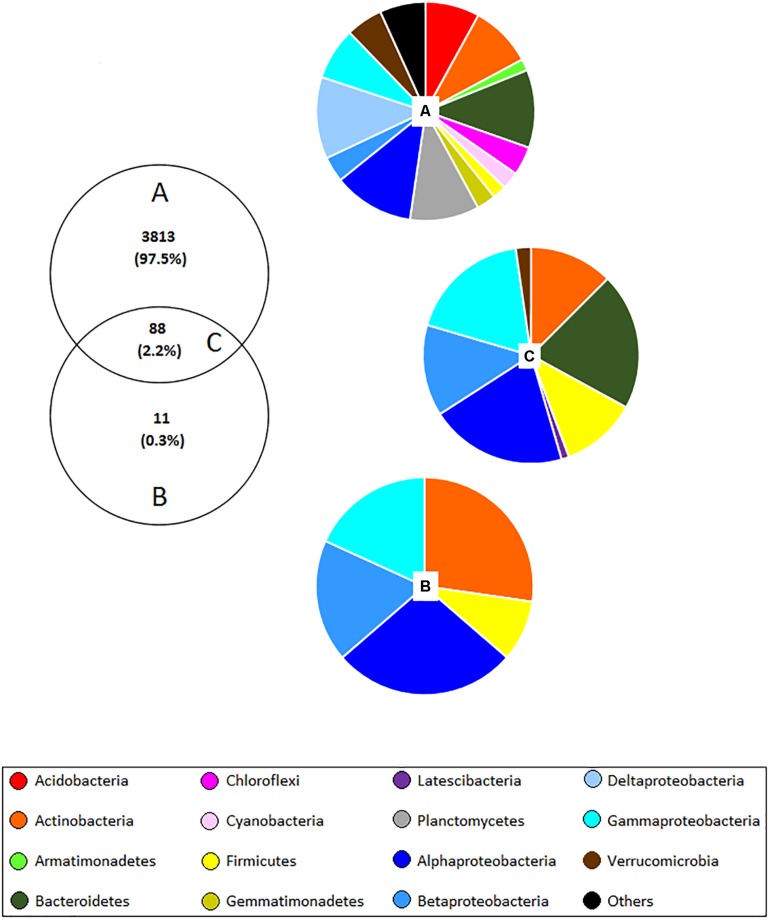
Venn diagram showing the number and proportion of shared OTUs **(C)** (at 97% similarity) detected with both unculturable **(A)** and culturable **(B)** methods in the wheat rhizosphere. Pie charts correspond to the percentage of bacterial phyla and classes of Proteobacteria assigned to each OTU. “Others” include 26 phyla corresponding to <1%, which include: BJ-169, BRC1, Candidatus Berkelbacteria, Chlamydiae, Chlorobi, Deinococcus–Thermus, Elusimicrobia, FBP, FCPU, Fibrobacteres, Gracilibacteria, Ignavibacteriae, Latescibacteria, Microgenomates, Nitrospirae, Omnitrophica, Parcubacteria, Peregrinibacteria, Saccharibacteria, Spirochaetae, SR1, Tectomicrobia, Tenericutes, TM6, WS2, and WWE3.

The culture-independent approach identified a total of 3,901 OTUs, whereas the culture-dependent method detected only 99 OTUs. 88 of these OTUs were found using both methods (Figure 3) indicating that 11 OTUs were absent in the culture-independent dataset; however, no significant differences were observed between samples obtained from different land management. Most of the unculturable OTUs that were previously flagged up as significantly different in this work were not observed with the culture-dependent method, thus new culturing media for isolation of these microbes should be developed. Concerning the common OTUs detected with both methods, 52.3% were assigned to Proteobacteria, 20.5% to Bacteroidetes, 12.5% to Actinobacteria, 11.4% to Firmicutes, 2.3% to Verrucomicrobia, and 1.1% to Latescibacteria. Besides, most of the OTUs which were assigned to genera have been reported in wheat rhizospheres such as *Achromobacter*, *Acinetobacter*, *Aeromonas*, *Agromyces*, *Bacillus*, *Brevundimonas*, *Cellvibrio*, *Chryseobacterium*, *Duganella*, *Dyadobacter*, *Flavobacterium*, *Klebsiella*, *Luteibacter*, *Lysobacter*, *Massilia*, *Microbacterium*, *Mucilaginibacter*, *Paenibacillus*, *Pedobacter*, *Pseudomonas*, *Pseudoxanthomonas*, *Rhizobium*, *Rhodanobacter*, *Rhodococcus*, *Serratia*, *Sphingomonas*, *Stenotrophomonas*, *Streptomyces*, and *Variovorax.*

### Land Management Effect on Rhizosphere and Endosphere Bacterial Community Assembly

In order to determine whether root compartment affected the culture-dependent bacterial community structure, NMDS plots of bacterial taxonomic composition of wheat rhizosphere and endosphere were constructed (Supplementary Figure S2). It was found that samples could be discriminated by wheat compartment, and land management had no effect on community selection (Supplementary Figure S2) (two-way PERMANOVA, plant compartment: *F* = 5.8452, *p* = 0.0001; land management: *F* = 0.6779, *p* = 0.4059).

From the OTUs isolated from the wheat rhizosphere and endosphere samples, a total of 12 genera were enriched in the rhizosphere. Two of these were representative of the Alphaproteobacteria (*Asticcacaulis and Caulobacter*), four of the Betaproteobacteria (*Burkholderia–Paraburkholderia*, *Duganella* and *Massilia*), one Gammaproteobacteria representative (*Stenotrophomonas*), three from the Bacteroidetes (*Chryseobacterium*, *Flavobacterium*, and *Pedobacter*), one Firmicutes (*Paenibacillus*), and one from the Actinobacteria (*Pseudarthrobacter*). Only two genera were found to be more abundant in the endosphere compartment and they were both representative of the Gammaproteobacteria (*Pseudomonas* and *Serratia*).

### Effect of the Seed Load on Rhizosphere Bacterial Community

**FIGURE 4:**
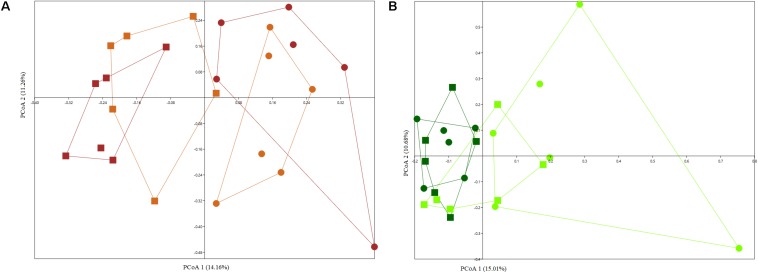
PCoA plots based on Jaccard distance matrix of culturable bacterial communities from the rhizosphere **(A)** and endosphere **(B)** of wheat grown in continuous arable or bare fallow soil. The percentage shown on each axis corresponds to the proportion of variation explained. Solid squares represent continuous arable soil and solid circles represent bare fallow soil. Light brown color represents wheat rhizosphere samples derived from the culturing of excised embryos and brown color indicates samples from the rhizosphere of complete seed-derived wheat plants. Dark green color represents samples obtained from the endosphere of complete seed-derived wheat plants and light green color represents samples from endosphere of wheat plants generated from excised embryos.

As expected plant compartment played a significant role in structuring culturable bacterial communities (Supplementary Figures S3A,B), with two separate clusters forming for rhizosphere and endosphere samples grown in arable and bare fallow soil, respectively, regardless of whether plants were derived from complete seeds or excised embryos (Arable soil – PERMANOVA, *F* = 2.953, *p* = 0.0001; Bare fallow soil – PERMANOVA, *F* = 2.985, *p* = 0.0001). When analyzing wheat rhizosphere, microbial seed load had no significant effect on culturable bacterial communities (Figure 4A) and the effect of land management was evident (two-way PERMANOVA, land management: *F* = 2.8559, *p* = 0.0001; seed load: *F* = 1.1291, *p* = 0.2653). On the other hand, seed load was important in shaping bacterial communities from the endosphere, with soil management being a secondary and less important factor (Figure 4B) (two-way PERMANOVA, land management: *F* = 1.5614, *p* = 0.0138; seed load: *F* = 1.8436, *p* = 0.0004).

The root endosphere of samples collected in bare fallow soil had a different compositional structure with 14 genera found to be differentially abundant when comparing entire seed and excised embryo generated wheat plants. Wheat plants generated from excised embryos had a higher abundance of *Chryseobacterium*, *Dyadobacter*, *Sphingomonas*, *Devosia*, *Caulobacter*, *Phenylobacterium*, *Novosphingobium*, *Rhizobium*, and *Bacillus*, whereas complete seed-derived endosphere samples had a significantly higher abundance of bacteria assigned to *Chitinophaga*, *Pedobacter*, *Flavobacterium*, *Pantoea*, and *Rheinheimera.* On the other hand, for bacteria from the endosphere of wheat grown in arable soil, only two genera were found to be significantly more abundant in complete seed-derived wheat plants (*Xanthomonas* and *Paenarthrobacter*) and one genus – *Chryseobacterium*, was more abundant in the endosphere of wheat plants generated from excised embryos.

## Discussion

One of the goals of this study was to use the Highfield experiment at Rothamsted to test the validity of culture-dependent and culture-independent approaches for studying the soil and root microbiome.

As expected, the total community analysis was able to identify a far greater number of OTUs compared to our culture method, and this was apparent as the culture-based methods could only distinguish the rhizosphere effect but not land management effect. In contrast, culture-independent analysis discriminated bacterial communities by niche and land managment treatment. This approach also detected differences in bulk soil communities over time, indicating a drift in selection of the soil microbiome in the conversion plots. However, it was intriguing to find that some OTUs detected in the more limited culture-based approach were absent from the total community method, highlighting that although the latter has a far higher resolution, it is insufficient to capture the entire microbial community even when using an average of 53,925 reads per sample. This is likely to be due to culture amplification bias, where particular microbes grow preferentially on a given medium and also PCR bias where some OTUs are poorly amplified by “universal” primers (Thijs et al., 2017), however, as both sample types used the same primers for amplification, this is not the likely explanation. Alternatively, this could also be due to these particular microbes being resistant to the soil DNA extraction protocol, or perhaps culture contamination, though their identification as typical soil organisms, such as members belonging to *Xanthomonas*, *Herbaspirillum*, *Rhodobacter*, *Phycicoccus*, *Curtobacterium*, *Phyllobacterium*, *Sanguibacter*, Phyllobacteriaceae, and the Planococcaceae family make the latter explanation unlikely. Besides, the culture-based method enabled isolation of bacteria commonly found in wheat rhizosphere which have also been detected with the 16S rRNA gene amplicon method (Rana et al., 2011; Turner et al., 2013; Yin et al., 2013; Gontia-Mishra et al., 2017; Granzow et al., 2017; Mahoney et al., 2017; Uksa et al., 2017; Flores-Núñez et al., 2018; Kumar et al., 2018; Araujo et al., 2019).

Although total community methods are useful to accurately describe the plant and soil microbiome, it is likely that in order to apply beneficial microbes to sustainable agricultural systems that they are amenable to culture. Recent advances in culture-based techniques for microbiomes have been developed, this is exemplified by the Ichip system (Nichols et al., 2010) which does not rely on standard culture media, and in the case of soil, it utilizes a dilution to extinction approach and immersion of diluted samples into the original soil substrate separated by a semi-permeable membrane. This allows the diffusion of nutrients into growth chambers and the culture of microbes under bespoke conditions. This method has dramatically increased the ability to culture the microbiome, but its usefulness to culture organisms in the necessary quantities for use as microbial inoculants is yet to be achieved. However, Bai et al. (2015) demonstrated that the majority of leaf and root-dwelling microbes of *Arabidopsis* were amenable to culture, suggesting that plant associated microbes are more accessible to culture than bulk soil specialists, and as such their exploitation in sustainable agriculture shows promise.

Our study also investigated the influence of plant root niche compartment as we examined in a culture-dependent manner both rhizosphere and root endosphere communities. We were unable to examine the culture-independent endosphere using our methodology as the 16S rRNA gene primers are also homologous to plant plastid sequences which are in far greater abundance than the microbial sequences in a given sample. Nevertheless, we were able to detect shifts in community structure based on niche, as indicated by enrichment of Bacterdoidetes in the rhizosphere. A relatively low proportional abundance of Bacteroidetes in the wheat endosphere has previously been reported under high N fertilization conditions (Robinson et al., 2016a). It is unknown why the Bacteroidetes are less competitive in this niche, especially as they have been isolated from the wheat rhizosphere (Robinson et al., 2016a) and are found in the endosphere of other plant species (Fitzpatrick et al., 2017) with a high overall relative abundance of ∼10% (Liu et al., 2017). It could be a matter of competitive exclusion by other members of the plant microbiome, a gating mechanism which precludes their colonization (Liu et al., 2017), the pH inside wheat roots not permitting growth of these bacteria, or perhaps a combination of these effects and other edaphic and environmental factors (Liu et al., 2017).

When analyzing culturable bacterial communities in plants grown in soil with different land managements (continuous arable, bare fallow, and grassland), no detectable differences between endosphere communities were observed. This is unsurprising due to the limited resolution of the culture-based method, and the fact that we were unable to detect differences in the rhizospheres of plants grown under these differing management regimes using a culture-dependent approach. Improved methods for culture-independent analysis of the wheat endosphere microbiome are needed: these have been successful with other plant hosts (Fitzpatrick et al., 2017; Zhao et al., 2017). However, the development of blocking primers to exclude plastid gene amplification, or other plastid exclusion methods such as density gradient centrifugation (Jiao et al., 2006) or the use of other non-plastid bacterial genes as targets for PCR such as *gyrB* could be used to test whether this is also the case with a culture-independent analysis of these samples.

For the “seed–embyo excision” experiment, a clear distinction between rhizosphere and endosphere culturable bacterial communities was observed which supported our own findings from field grown plants in this work. van Overbeek et al. (2011) suggested there might be a major role played by the mother plant in the recruitment of the endosphere microbiome. Rhizosphere recruitment is known to be partly due to the release of plant exudates and it is possible that this may also be the case for the endosphere (Bulgarelli et al., 2013).

Another goal of this study was to evaluate the importance of microbial seed load on rhizosphere and endosphere wheat microbiome assembly. We have previously found that wheat embryos excised from seeds are free of the seed borne microbiome (Robinson et al., 2016b). When using a culture-dependent method, microbial seed load was found to not be important for construction of the rhizosphere compartment, indicating that rhizosphere competent microbes are readily available to colonize and select the rhizosphere microbiome in soil from contrasting land managements. This once more highlights the ability of plants to shape their microbial communities (Sasse et al., 2018).

We also found that the wheat endosphere microbiomes resulting from the culture of entire seeds and microbe free excised embryos in wheat adapted soil could not be discrimminated. However, when this planting regime was performed in bare fallow soil the root endosphere microbiomes of complete seed and excised embryo derived plants could be clearly distinguished. This implies that the microbial reservoir of the bare fallow soil tested in this work impaired the ability of the plants generated from microbe-free embryos to construct a “normal” root endosphere microbiome to a greater extent than the rhizosphere microbiome. It seems likely that the microbes required to form the “normal” endosphere are absent or in reduced abundance in bare fallow soil relative to wheat adapted arable soil. The results for culture-independent analysis of rhizosphere microbiomes demonstrated an effect of land use, which was undetectable when using a culture-dependent method on the same samples. As such, our ability to detect differences in the wheat root endosphere microbiome, even when using the relatively low resolution culture-dependent approach implies that microbial seed load is intimately associated with the development of the root endosphere microbiome to a greater extent than the rhizosphere microbiome. These findings support previous work that seeds are not sterile and that microbes can be vertically transmitted (Berg and Raaijmakers, 2018).

The results presented in this work support and extend previous work at the Rothamsted Highfield experimental site (Hirsch et al., 2017), which considered only the bulk soil, and they increase our confidence in the robustness of soil microbiology methodology in the high-throughput sequencing era. Clear distinctions between the wheat rhizosphere and bulk soil microbiomes were found and this has also been reported for other crops, such as maize (Yang et al., 2017), barley (Bulgarelli et al., 2015), soybean (Mendes et al., 2014), rice (Edwards et al., 2015), sorghum (Schlemper et al., 2017), and common bean (Pérez-Jaramillo et al., 2017). It is recognized that soil type has great influence on the structure of bacterial communities (Kuramae et al., 2012) and although these previous studies used different soil types, the similarities and differences observed with other plant hosts reported in the literature describe to what extent the selection of the plant microbiome varies between crop hosts. Finally, the use of an embryo excision method facilitates studies into the microbial transmission from seeds to plants. Taken together, these findings provide information for future studies toward the exploitation of the plant microbiome for sustainable crop production.

## Data Availability Statement

The datasets analyzed during the current study are available in the NCBI Sequence Read Archive (SRA), accession PRJNA579554.

## Author Contributions

TM, PH, and IC designed the experiments. TM, RH, RR, and IC performed the experiments and collected the data. TM and VK analyzed the data. DH developed an automatic script for running QIIME analyses. VK and TM wrote the manuscript. TM, PH, RM, VK, and MR edited and commented on the manuscript.

## Conflict of Interest

The authors declare that the research was conducted in the absence of any commercial or financial relationships that could be construed as a potential conflict of interest.

## Footnotes

1 www.drive5.com/usearch

## Supplementary Material

FIGURE S1 Extended error plots showing the log-transformed abundance of sequences that were statistically different (*p* < 0.05) at phylum level when comparing bulk soil (dark gray) and rhizosphere (light gray) of wheat grown in Highfield.

FIGURE S2 2D-NMDS plot based on Jaccard distance matrix of culturable wheat rhizosphere (circles) and endosphere (squares) bacterial community of wheat obtained from Highfield experiment under three land managements: continuous arable (yellow), conversion of bare fallow to arable (brown), and conversion of grassland to arable (green).

FIGURE S3 PCoA plots based on Jaccard distance matrix of culturable bacterial communities from bulk soil, rhizosphere, and endosphere of wheat grown in continuous arable **(A)** or bare fallow **(B)** soil. The percentage shown on each axis corresponds to the proportion of variation explained. Solid squares represent continuous arable soil and solid circles represent bare fallow soil. Red color indicates samples from bulk soil, dark red color indicates samples from wheat rhizosphere, and dark green color represents samples collected from the wheat endosphere.

FIGURE S4 Heatmap showing the 60 significantly differentially abundant OTUs detected using DESeq2 among different land management treatments [continuous arable (AA), conversion of bare fallow to arable (BA), and conversion of grassland to arable (GA)]. The color scheme varies from light gray to black, with light gray color indicating OTUs which were found to be less abundant, and dark gray and black indicating which OTUs were enriched, with black being more abundant than those indicated by the dark gray color.

TABLE S1 Sample IDs and information.
