# Supplementary figures and images for: Land Management and Microbial Seed Load Effect on Rhizosphere and Endosphere Bacterial Community Assembly in Wheat
Source: Front Microbiol. 2019 Nov 15;10:2625. doi: 10.3389/fmicb.2019.02625 (PMC6873152; doi:10.3389/fmicb.2019.02625)

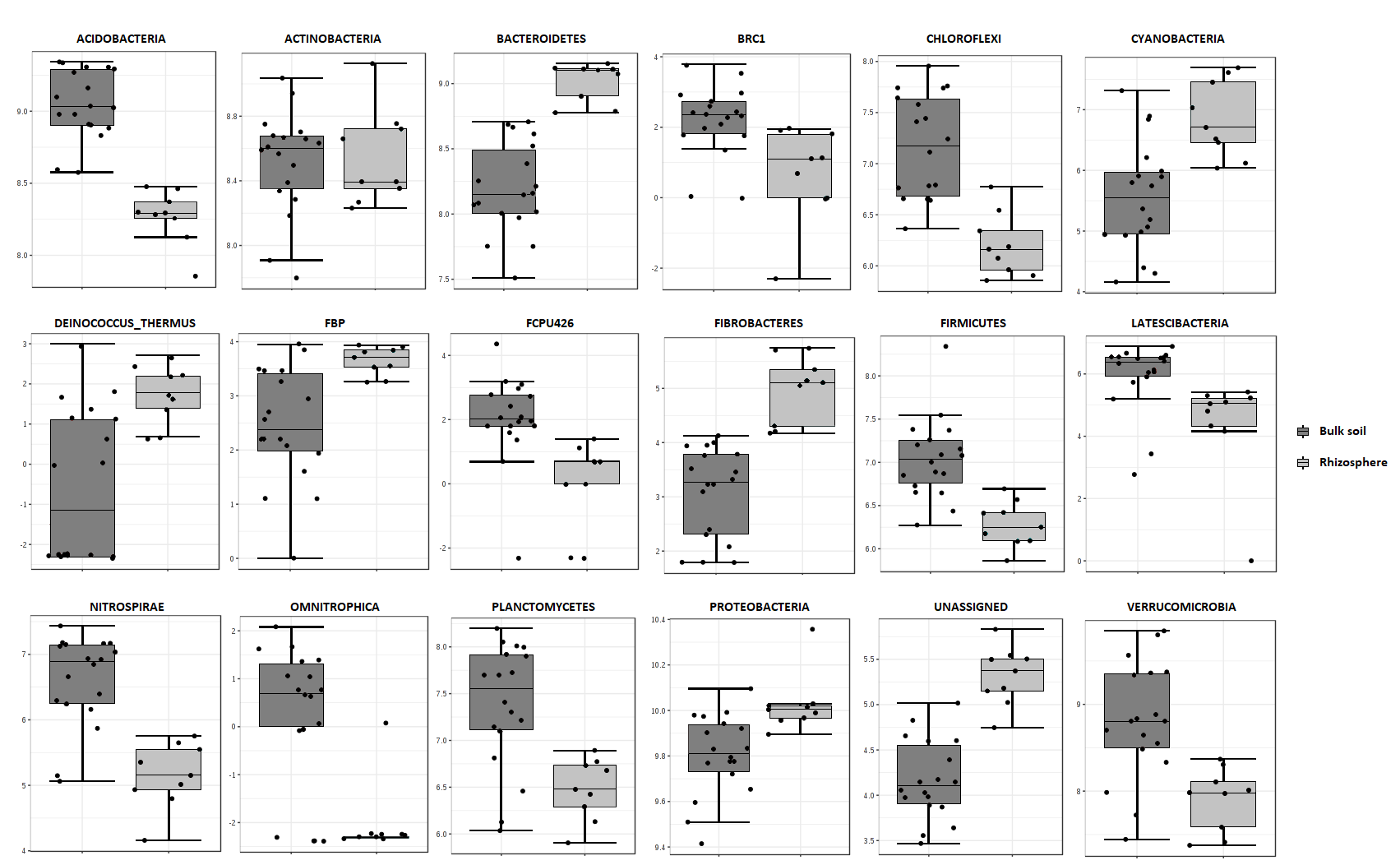

Supplement: FIGURE S1 — Extended error plots showing the log-transformed abundance of sequences that were statistically different (p < 0.05) at phylum level when comparing bulk soil (dark gray) and rhizosphere (light gray) of wheat grown in Highfield. [file Image_1.TIF]

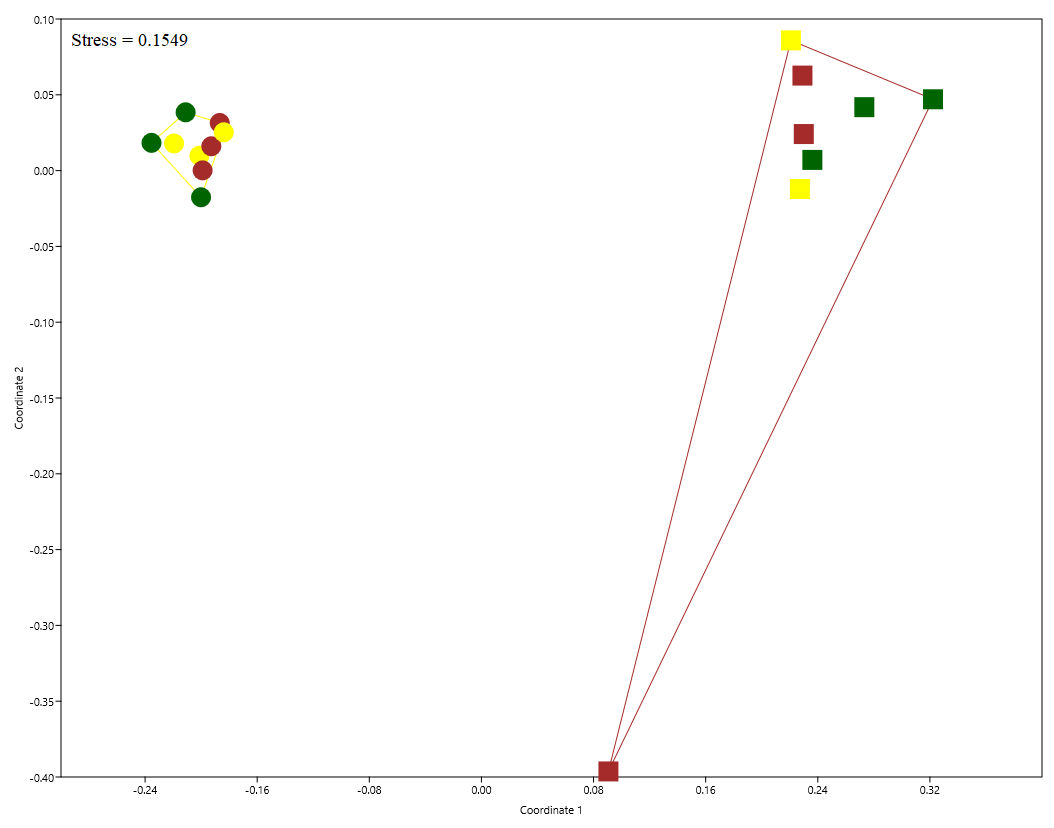

Supplement: FIGURE S2 — 2D-NMDS plot based on Jaccard distance matrix of culturable wheat rhizosphere (circles) and endosphere (squares) bacterial community of wheat obtained from Highfield experiment under three land managements: continuous arable (yellow), conversion of bare fallow to arable (brown), and conversion of grassland to arable (green). [file Image_2.TIF]

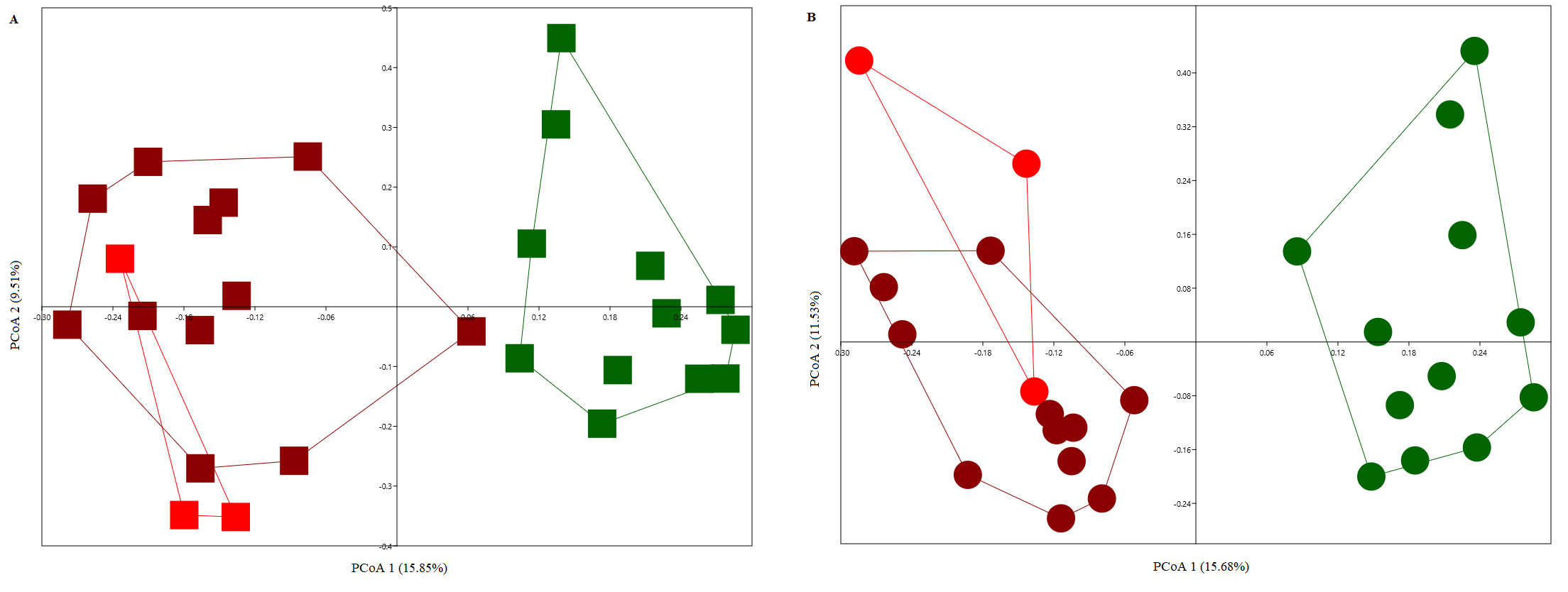

Supplement: FIGURE S3 — PCoA plots based on Jaccard distance matrix of culturable bacterial communities from bulk soil, rhizosphere, and endosphere of wheat grown in continuous arable (A) or bare fallow (B) soil. The percentage shown on each axis corresponds to the proportion of variation explained. Solid squares represent continuous arable soil and solid circles represent bare fallow soil. Red color indicates samples from bulk soil, dark red color indicates samples from wheat rhizosphere, and dark green color represents samples collected from the wheat endosphere. [file Image_3.TIF]

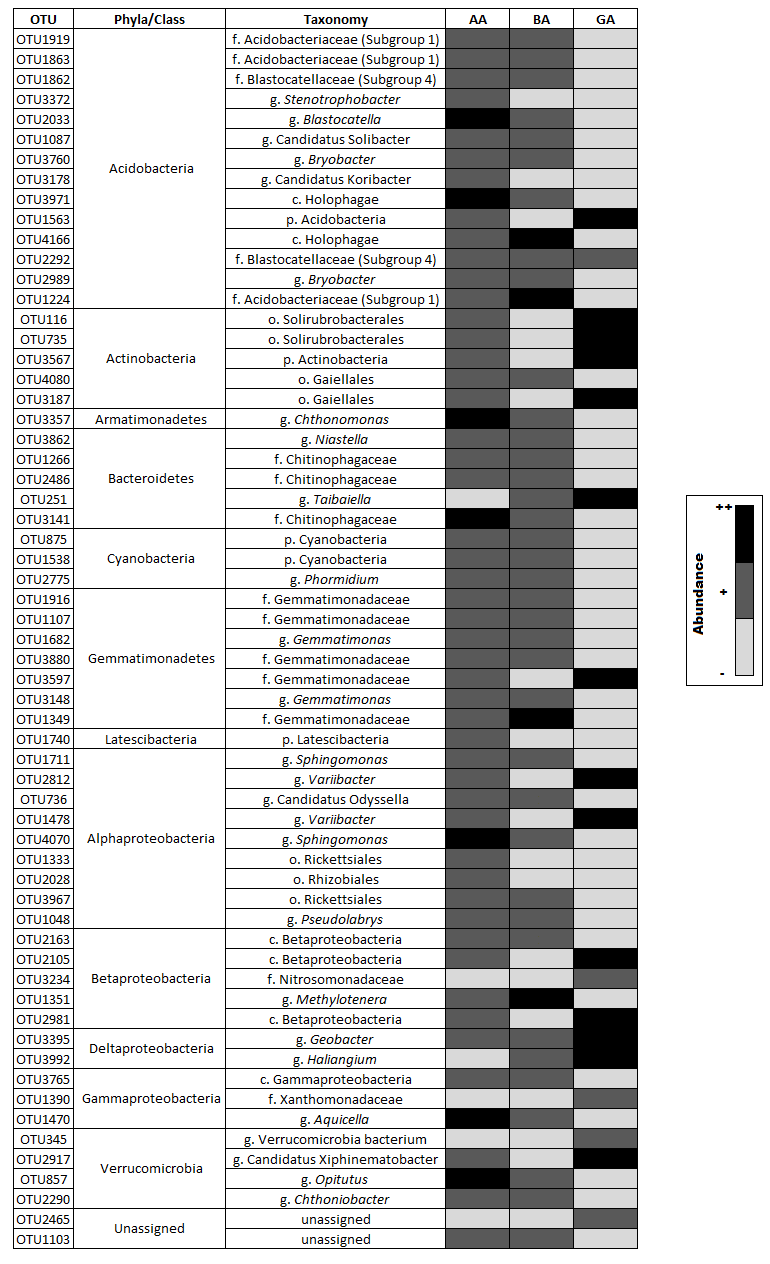

Supplement: FIGURE S4 — Heatmap showing the 60 significantly differentially abundant OTUs detected using DESeq2 among different land management treatments [continuous arable (AA), conversion of bare fallow to arable (BA), and conversion of grassland to arable (GA)]. The color scheme varies from light gray to black, with light gray color indicating OTUs which were found to be less abundant, and dark gray and black indicating which OTUs were enriched, with black being more abundant than those indicated by the dark gray color. [file Image_4.PNG]
